# Bis[2-(4-benzo­yloxy-2-hy­droxy­benzo­yl)-1-phenyl­ethenolato]diethano­lzinc(II)

**DOI:** 10.1107/S1600536810036202

**Published:** 2010-09-25

**Authors:** Kai Dong, Juan Sun, Ban-Feng Ruan, Hai-Bin Gong

**Affiliations:** a State Key Laboratory of Pharmaceutical Biotechnology, Nanjing University, Nanjing 210093, People’s Republic of China; b Xuzhou Central Hospital, Xuzhou Cardiovascular Disease Institute, Xuzhou 221009, Jiangsu, People’s Republic of China

## Abstract

The mononuclear title complex, [Zn(C_22_H_15_O_5_)_2_(C_2_H_5_OH)_2_], contains a Zn^II^ atom (site symmetry 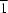) surrounded by six O atoms of the keto groups of two substituted 1,3-diketonate ligands and of two ethanol mol­ecules, resulting in a distorted octa­hedral coordination environment. The mol­ecular configuration is stabilized by an intra­molecular hydrogen bond between the phenolic hy­droxy group and the adjacent keto group. The hy­droxy group acts likewise as an acceptor of an inter­molecular O—H⋯O hydrogen bond with the hy­droxy group of the ethanol mol­ecule as the donor. The hydrogen-bonding scheme leads to the formation of supra­molecular layers parallel to (010).

## Related literature

For the role of zinc in enzymes and in bioinorganic chemistry, see: Bertini *et al.* (1994 ▶); Lipscomb & Strater (1996 ▶); Vallee & Auld (1993 ▶); Zhu *et al.* (2003 ▶).
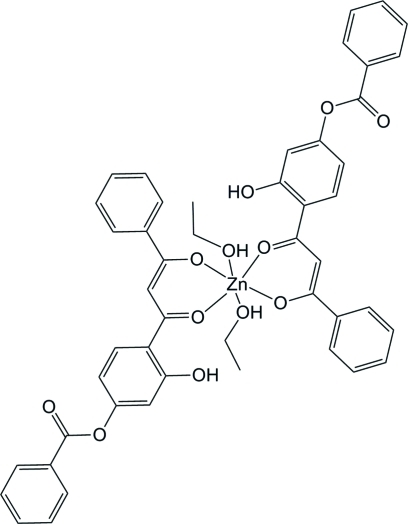


## Experimental

### 

#### Crystal data

[Zn(C_22_H_15_O_5_)_2_(C_2_H_6_O)_2_]
*M* *_r_* = 876.19
Triclinic, 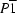

*a* = 7.170 (5) Å
*b* = 9.399 (5) Å
*c* = 16.457 (5) Å
α = 106.590 (5)°
β = 95.596 (5)°
γ = 90.989 (5)°
*V* = 1056.6 (10) Å^3^
*Z* = 1
Mo *K*α radiation
μ = 0.65 mm^−1^
*T* = 298 K
0.30 × 0.20 × 0.20 mm

#### Data collection

Bruker SMART CCD area-detector diffractometer
Absorption correction: multi-scan (*SADABS*; Bruker, 2000 ▶) *T*_min_ = 0.830, *T*_max_ = 0.882
6185 measured reflections
4255 independent reflections
2898 reflections with *I* > 2σ(*I*)
*R*_int_ = 0.017

#### Refinement

*R*[*F* ^2^ > 2σ(*F* ^2^)] = 0.050
*wR*(*F* ^2^) = 0.142
*S* = 1.02
4255 reflections
279 parameters
14 restraints
H-atom parameters constrained
Δρ_max_ = 0.80 e Å^−3^
Δρ_min_ = −0.49 e Å^−3^

### 

Data collection: *SMART* (Bruker, 2000 ▶); cell refinement: *SAINT* (Bruker, 2000 ▶); data reduction: *SAINT*; program(s) used to solve structure: *SHELXS97* (Sheldrick, 2008 ▶); program(s) used to refine structure: *SHELXL97* (Sheldrick, 2008 ▶); molecular graphics: *SHELXTL* (Sheldrick, 2008 ▶); software used to prepare material for publication: *SHELXTL*.

## Supplementary Material

Crystal structure: contains datablocks global, I. DOI: 10.1107/S1600536810036202/wm2402sup1.cif

Structure factors: contains datablocks I. DOI: 10.1107/S1600536810036202/wm2402Isup2.hkl

Additional supplementary materials:  crystallographic information; 3D view; checkCIF report

## Figures and Tables

**Table 1:** Selected bond lengths (Å)

Zn1—O5^i^	2.002 (2)
Zn1—O4^i^	2.034 (2)
Zn1—O6^i^	2.203 (3)

Symmetry code: (i) 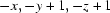.

**Table 2:** Hydrogen-bond geometry (Å, °)

*D*—H⋯*A*	*D*—H	H⋯*A*	*D*⋯*A*	*D*—H⋯*A*
O3—H3⋯O4	0.82	1.74	2.470 (3)	147
O6—H10*A*⋯O3^ii^	0.86	2.04	2.824 (4)	151

Symmetry code: (ii) 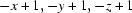.

**Fig. 1.**
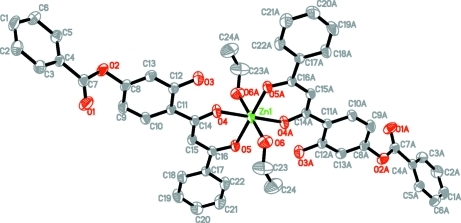
The structure and atom-numbering scheme of the title compound (I), showing 30% probability displacement ellipsoids. [Symmetry code: (A) -x, -y+1, -z+1.]

**Fig. 2.**
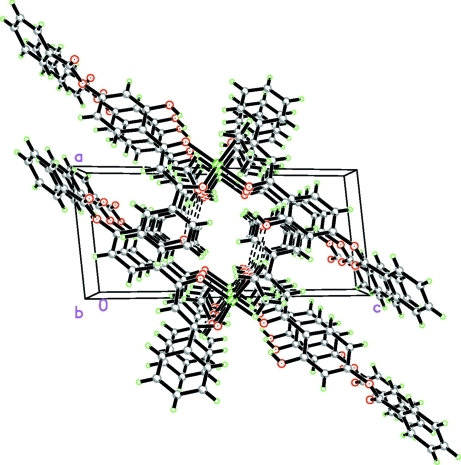
The crystal packing of the title compound viewed along the b axis. Hydrogen bonding interactions are indicated by dashed lines.

## supplementary crystallographic information

### Comment

Zinc, the second most abundant transition metal in biology, functions as the active site of hydrolytic enzymes, such as carboxypeptidase and carbonic anhydrase, where it is in a hard donor coordination environment of nitrogen and oxygen (Lipscomb & Strater, 1996; Bertini *et al.*, 1994). Zinc has long been recognized as an important cofactor in biological molecules, either as a structural template in protein folding or as a Lewis acid catalyst that can readily adopt 4-, 5- or 6-coordination (Vallee & Auld, 1993). Studies of the structures and properties of the metal coordination sites in in zinc enzymes recently were in focus in the field of bioinorganic chemistry (Zhu *et al.*, 2003). A couple of chemical systems have been developed to reproduce the zinc coordination core. On our continuation of the research in this field, the crystal structure of a mononuclear zinc(II) compound, (I), is reported here.

In the structure of (I), the Zn^II^ atom is situated on an inversion center and adopts a slightly distorted octahedral coordination (Fig. 1). It is surrounded by six O atoms from the keto groups of two 1,3-diketonate ligands and by two ethanol molecules. The Zn^II^ atom and the four O atoms from the diketone ligands form the equatorial plane with similar Zn—O bond lengths of 2.002 (2) and 2.034 (2) Å. The two ethanol O atoms are in the axial positions with considerably longer Zn—O bond lengths of 2.203 (3) Å (Table 1). The two trans-angles at the zinc(II) center are 180° by symmetry; all angles around Zn1 are close to 90°, ranging from 85.93 (11) to 94.07 (11)°. In the crystal structure, intramolecular and intermolecular O—H···O hydrogen bonding leads to a layered assembly parallel to (010) (Table 2; Fig. 2).

### Experimental

To a ethanol solution (15 ml) of 3-hydroxy-4-(3-oxo-3-phenylpropanoyl)phenyl benzoic acid (0.36 g, 0.001mol), anhydrous zinc acetate (0.092 g, 0.0005 mol) was added at room temperature. After addition, the reaction mixture was refluxed for 30 min with stirring and then was filtered. Single crystals of the title compound were obtained by slow evaporation of the filtrate.

### Refinement

All hydrogen atoms were placed in geometrically idealized positions and constrained to ride on their parent atoms, with C—H = 0.93, 0.96(–CH_3_), 0.97 (–CH_2_), 0.86 (–OH) Å, *U*_iso_(H) = 1.2 *U*_eq_ (C or –OH), 1.5 *U*_eq_ (–CH_3_), respectively.

### Crystal data

**Table:**

[Zn(C_22_H_15_O_5_)_2_(C_2_H_6_O)_2_]	*Z* = 1
*M**_r_* = 876.19	*F*(000) = 456
Triclinic, *P*1	*D*_x_ = 1.377 Mg m^−^^3^
Hall symbol: -P 1	Mo *K*α radiation, λ = 0.71069 Å
*a* = 7.170 (5) Å	Cell parameters from 2800 reflections
*b* = 9.399 (5) Å	θ = 2.5–26.2°
*c* = 16.457 (5) Å	µ = 0.65 mm^−^^1^
α = 106.590 (5)°	*T* = 298 K
β = 95.596 (5)°	Block, colourless
γ = 90.989 (5)°	0.30 × 0.20 × 0.20 mm
*V* = 1056.6 (10) Å^3^	

### Data collection

**Table:**

Bruker SMART CCD area-detector diffractometer	4255 independent reflections
Radiation source: fine-focus sealed tube	2898 reflections with *I* > 2σ(*I*)
graphite	*R*_int_ = 0.017
φ and ω scans	θ_max_ = 26.5°, θ_min_ = 2.3°
Absorption correction: multi-scan (*SADABS*; Bruker, 2000)	*h* = −9→8
*T*_min_ = 0.830, *T*_max_ = 0.882	*k* = −11→11
6185 measured reflections	*l* = −18→20

### Refinement

**Table:**

Refinement on *F*^2^	Primary atom site location: structure-invariant direct methods
Least-squares matrix: full	Secondary atom site location: difference Fourier map
*R*[*F*^2^ > 2σ(*F*^2^)] = 0.050	Hydrogen site location: inferred from neighbouring sites
*wR*(*F*^2^) = 0.142	H-atom parameters constrained
*S* = 1.02	*w* = 1/[σ^2^(*F*_o_^2^) + (0.0597*P*)^2^ + 0.7172*P*] where *P* = (*F*_o_^2^ + 2*F*_c_^2^)/3
4255 reflections	(Δ/σ)_max_ < 0.001
279 parameters	Δρ_max_ = 0.80 e Å^−^^3^
14 restraints	Δρ_min_ = −0.49 e Å^−^^3^

### Special details

**Table:**

Geometry. All esds (except the esd in the dihedral angle between two l.s. planes) are estimated using the full covariance matrix. The cell esds are taken into account individually in the estimation of esds in distances, angles and torsion angles; correlations between esds in cell parameters are only used when they are defined by crystal symmetry. An approximate (isotropic) treatment of cell esds is used for estimating esds involving l.s. planes.
Refinement. Refinement of F^2^ against ALL reflections. The weighted R-factor wR and goodness of fit S are based on F^2^, conventional R-factors R are based on F, with F set to zero for negative F^2^. The threshold expression of F^2^ > 2sigma(F^2^) is used only for calculating R-factors(gt) etc. and is not relevant to the choice of reflections for refinement. R-factors based on F^2^ are statistically about twice as large as those based on F, and R- factors based on ALL data will be even larger.

### Fractional atomic coordinates and isotropic or equivalent isotropic displacement parameters (Å^2^)

**Table:**

	*x*	*y*	*z*	*U*_iso_*/*U*_eq_	
C1	1.0307 (7)	0.0321 (6)	−0.1364 (3)	0.0787 (14)	
H1	1.1025	−0.0287	−0.1751	0.094*	
C2	1.0779 (7)	0.1805 (6)	−0.1042 (3)	0.0828 (14)	
H2	1.1814	0.2205	−0.1214	0.099*	
C3	0.9730 (6)	0.2707 (5)	−0.0466 (3)	0.0670 (11)	
H3A	1.0040	0.3720	−0.0260	0.080*	
C4	0.8221 (5)	0.2120 (4)	−0.0193 (2)	0.0465 (8)	
C5	0.7732 (5)	0.0614 (4)	−0.0524 (2)	0.0572 (10)	
H5	0.6708	0.0209	−0.0347	0.069*	
C6	0.8769 (7)	−0.0277 (5)	−0.1115 (3)	0.0723 (12)	
H6	0.8431	−0.1283	−0.1345	0.087*	
C7	0.7210 (5)	0.3140 (5)	0.0470 (2)	0.0522 (9)	
C8	0.4913 (5)	0.3187 (4)	0.1426 (2)	0.0533 (9)	
C9	0.3464 (5)	0.4014 (5)	0.1234 (2)	0.0625 (11)	
H9	0.3287	0.4179	0.0701	0.075*	
C10	0.2282 (5)	0.4591 (4)	0.1841 (2)	0.0560 (10)	
H10	0.1290	0.5137	0.1707	0.067*	
C11	0.2513 (4)	0.4390 (4)	0.2655 (2)	0.0417 (8)	
C12	0.4069 (5)	0.3582 (4)	0.2832 (2)	0.0462 (8)	
C13	0.5241 (5)	0.2980 (4)	0.2214 (2)	0.0539 (9)	
H13	0.6247	0.2436	0.2336	0.065*	
C14	0.1192 (4)	0.4955 (4)	0.3303 (2)	0.0427 (8)	
C15	−0.0393 (5)	0.5691 (4)	0.3129 (2)	0.0475 (8)	
H15	−0.0592	0.5804	0.2584	0.057*	
C16	−0.1725 (4)	0.6280 (4)	0.3689 (2)	0.0409 (7)	
C17	−0.3308 (4)	0.7128 (4)	0.3413 (2)	0.0425 (8)	
C18	−0.3685 (5)	0.7198 (5)	0.2587 (2)	0.0648 (11)	
H18	−0.2969	0.6672	0.2169	0.078*	
C19	−0.5106 (6)	0.8032 (6)	0.2374 (3)	0.0817 (14)	
H19	−0.5329	0.8071	0.1815	0.098*	
C20	−0.6184 (6)	0.8797 (6)	0.2966 (3)	0.0816 (14)	
H20	−0.7108	0.9394	0.2824	0.098*	
C21	−0.5890 (7)	0.8675 (6)	0.3773 (3)	0.0971 (18)	
H21	−0.6660	0.9158	0.4179	0.117*	
C22	−0.4472 (6)	0.7849 (5)	0.3996 (3)	0.0749 (13)	
H22	−0.4299	0.7778	0.4551	0.090*	
C23	0.1763 (9)	0.8386 (7)	0.5795 (5)	0.132 (2)	
H23A	0.1946	0.8477	0.5236	0.159*	
H23B	0.2850	0.8879	0.6176	0.159*	
C24	0.0214 (9)	0.9216 (7)	0.6062 (5)	0.129 (2)	
H24A	−0.0890	0.8780	0.5689	0.193*	
H24B	0.0426	1.0218	0.6046	0.193*	
H24C	0.0044	0.9217	0.6634	0.193*	
O1	0.7452 (5)	0.4469 (3)	0.0716 (2)	0.0838 (10)	
O2	0.5993 (4)	0.2389 (3)	0.07885 (17)	0.0653 (7)	
O3	0.4461 (4)	0.3341 (4)	0.36033 (16)	0.0670 (8)	
H3	0.3672	0.3722	0.3917	0.100*	
O4	0.1634 (3)	0.4709 (3)	0.40277 (15)	0.0600 (7)	
O5	−0.1712 (3)	0.6190 (3)	0.44437 (14)	0.0547 (7)	
O6	0.1818 (4)	0.6942 (3)	0.5742 (2)	0.0773 (9)	
H10A	0.2735	0.6729	0.6057	0.093*	
Zn1	0.0000	0.5000	0.5000	0.0548 (2)	

### Atomic displacement parameters (Å^2^)

**Table:**

	*U*^11^	*U*^22^	*U*^33^	*U*^12^	*U*^13^	*U*^23^
C1	0.084 (3)	0.097 (4)	0.057 (3)	0.036 (3)	0.033 (2)	0.014 (3)
C2	0.083 (3)	0.093 (4)	0.089 (3)	0.017 (3)	0.055 (3)	0.035 (3)
C3	0.068 (3)	0.065 (3)	0.077 (3)	0.008 (2)	0.033 (2)	0.026 (2)
C4	0.0436 (19)	0.058 (2)	0.0402 (18)	0.0108 (17)	0.0117 (15)	0.0155 (17)
C5	0.051 (2)	0.063 (3)	0.057 (2)	0.0030 (19)	0.0140 (18)	0.0138 (19)
C6	0.078 (3)	0.066 (3)	0.063 (3)	0.013 (2)	0.014 (2)	0.002 (2)
C7	0.045 (2)	0.061 (3)	0.053 (2)	0.0063 (18)	0.0159 (16)	0.0166 (19)
C8	0.049 (2)	0.057 (2)	0.055 (2)	0.0120 (18)	0.0262 (17)	0.0105 (18)
C9	0.061 (2)	0.087 (3)	0.049 (2)	0.019 (2)	0.0232 (18)	0.027 (2)
C10	0.049 (2)	0.076 (3)	0.052 (2)	0.0238 (19)	0.0175 (16)	0.0276 (19)
C11	0.0332 (17)	0.050 (2)	0.0443 (18)	0.0100 (14)	0.0108 (13)	0.0142 (15)
C12	0.0352 (17)	0.059 (2)	0.0461 (19)	0.0112 (16)	0.0106 (14)	0.0148 (17)
C13	0.0413 (19)	0.062 (2)	0.060 (2)	0.0182 (17)	0.0158 (16)	0.0157 (19)
C14	0.0331 (17)	0.056 (2)	0.0419 (18)	0.0105 (15)	0.0102 (13)	0.0158 (16)
C15	0.0419 (19)	0.066 (2)	0.0399 (18)	0.0205 (17)	0.0128 (14)	0.0207 (17)
C16	0.0343 (17)	0.051 (2)	0.0398 (18)	0.0085 (15)	0.0068 (13)	0.0150 (15)
C17	0.0362 (17)	0.050 (2)	0.0434 (18)	0.0107 (15)	0.0061 (14)	0.0152 (15)
C18	0.058 (2)	0.096 (3)	0.053 (2)	0.033 (2)	0.0175 (18)	0.037 (2)
C19	0.075 (3)	0.121 (4)	0.066 (3)	0.046 (3)	0.010 (2)	0.050 (3)
C20	0.068 (3)	0.105 (4)	0.082 (3)	0.049 (3)	0.008 (2)	0.041 (3)
C21	0.088 (3)	0.139 (5)	0.073 (3)	0.078 (3)	0.027 (3)	0.034 (3)
C22	0.072 (3)	0.111 (4)	0.051 (2)	0.054 (3)	0.019 (2)	0.030 (2)
C23	0.112 (4)	0.086 (2)	0.197 (5)	0.020 (3)	−0.007 (4)	0.044 (3)
C24	0.114 (4)	0.110 (4)	0.147 (4)	0.040 (3)	0.014 (3)	0.009 (3)
O1	0.092 (2)	0.0581 (19)	0.098 (2)	0.0027 (17)	0.0523 (19)	0.0045 (17)
O2	0.0656 (17)	0.0624 (17)	0.0721 (18)	0.0123 (14)	0.0423 (14)	0.0140 (14)
O3	0.0497 (15)	0.107 (2)	0.0525 (15)	0.0400 (15)	0.0158 (12)	0.0316 (16)
O4	0.0456 (14)	0.100 (2)	0.0461 (14)	0.0360 (14)	0.0165 (11)	0.0350 (14)
O5	0.0496 (14)	0.0804 (18)	0.0429 (13)	0.0331 (13)	0.0165 (11)	0.0264 (13)
O6	0.0566 (17)	0.0793 (18)	0.092 (2)	0.0247 (15)	−0.0049 (15)	0.0215 (17)
Zn1	0.0475 (4)	0.0847 (5)	0.0425 (3)	0.0331 (3)	0.0159 (3)	0.0296 (3)

### Geometric parameters (Å, °)

**Table:**

C1—C2	1.366 (7)	C15—H15	0.9300
C1—C6	1.378 (6)	C16—O5	1.269 (4)
C1—H1	0.9300	C16—C17	1.506 (4)
C2—C3	1.373 (6)	C17—C22	1.372 (5)
C2—H2	0.9300	C17—C18	1.379 (5)
C3—C4	1.375 (5)	C18—C19	1.375 (5)
C3—H3A	0.9300	C18—H18	0.9300
C4—C5	1.389 (5)	C19—C20	1.352 (6)
C4—C7	1.489 (5)	C19—H19	0.9300
C5—C6	1.377 (5)	C20—C21	1.363 (6)
C5—H5	0.9300	C20—H20	0.9300
C6—H6	0.9300	C21—C22	1.374 (5)
C7—O1	1.202 (4)	C21—H21	0.9300
C7—O2	1.346 (4)	C22—H22	0.9300
C8—C13	1.365 (5)	C23—O6	1.337 (6)
C8—C9	1.374 (5)	C23—C24	1.402 (6)
C8—O2	1.411 (4)	C23—H23A	0.9700
C9—C10	1.372 (5)	C23—H23B	0.9700
C9—H9	0.9300	C24—H24A	0.9600
C10—C11	1.400 (5)	C24—H24B	0.9600
C10—H10	0.9300	C24—H24C	0.9600
C11—C12	1.414 (4)	O3—H3	0.8200
C11—C14	1.486 (4)	O4—Zn1	2.034 (2)
C12—O3	1.356 (4)	O5—Zn1	2.002 (2)
C12—C13	1.383 (5)	O6—Zn1	2.203 (3)
C13—H13	0.9300	O6—H10A	0.8600
C14—O4	1.289 (4)	Zn1—O5^i^	2.002 (2)
C14—C15	1.389 (4)	Zn1—O4^i^	2.034 (2)
C15—C16	1.402 (4)	Zn1—O6^i^	2.203 (3)
			
C2—C1—C6	120.2 (4)	C18—C17—C16	123.2 (3)
C2—C1—H1	119.9	C19—C18—C17	120.9 (4)
C6—C1—H1	119.9	C19—C18—H18	119.5
C1—C2—C3	120.2 (4)	C17—C18—H18	119.5
C1—C2—H2	119.9	C20—C19—C18	121.0 (4)
C3—C2—H2	119.9	C20—C19—H19	119.5
C2—C3—C4	120.4 (4)	C18—C19—H19	119.5
C2—C3—H3A	119.8	C19—C20—C21	118.7 (4)
C4—C3—H3A	119.8	C19—C20—H20	120.7
C3—C4—C5	119.4 (3)	C21—C20—H20	120.7
C3—C4—C7	117.8 (3)	C20—C21—C22	121.0 (4)
C5—C4—C7	122.8 (3)	C20—C21—H21	119.5
C6—C5—C4	119.8 (4)	C22—C21—H21	119.5
C6—C5—H5	120.1	C17—C22—C21	120.9 (4)
C4—C5—H5	120.1	C17—C22—H22	119.6
C5—C6—C1	119.9 (4)	C21—C22—H22	119.6
C5—C6—H6	120.0	O6—C23—C24	121.8 (6)
C1—C6—H6	120.0	O6—C23—H23A	106.9
O1—C7—O2	122.9 (3)	C24—C23—H23A	106.9
O1—C7—C4	125.4 (3)	O6—C23—H23B	106.9
O2—C7—C4	111.7 (3)	C24—C23—H23B	106.9
C13—C8—C9	121.4 (3)	H23A—C23—H23B	106.7
C13—C8—O2	117.3 (3)	C23—C24—H24A	109.5
C9—C8—O2	121.0 (3)	C23—C24—H24B	109.5
C10—C9—C8	118.9 (3)	H24A—C24—H24B	109.5
C10—C9—H9	120.6	C23—C24—H24C	109.5
C8—C9—H9	120.6	H24A—C24—H24C	109.5
C9—C10—C11	122.5 (3)	H24B—C24—H24C	109.5
C9—C10—H10	118.7	C7—O2—C8	119.2 (3)
C11—C10—H10	118.7	C12—O3—H3	109.5
C10—C11—C12	116.4 (3)	C14—O4—Zn1	126.7 (2)
C10—C11—C14	122.9 (3)	C16—O5—Zn1	126.6 (2)
C12—C11—C14	120.7 (3)	C23—O6—Zn1	132.2 (4)
O3—C12—C13	117.2 (3)	C23—O6—H10A	114.0
O3—C12—C11	121.8 (3)	Zn1—O6—H10A	113.8
C13—C12—C11	121.0 (3)	O5—Zn1—O5^i^	180.00 (11)
C8—C13—C12	119.7 (3)	O5—Zn1—O4	89.37 (9)
C8—C13—H13	120.1	O5^i^—Zn1—O4	90.63 (9)
C12—C13—H13	120.1	O5—Zn1—O4^i^	90.63 (9)
O4—C14—C15	123.3 (3)	O5^i^—Zn1—O4^i^	89.37 (9)
O4—C14—C11	115.0 (3)	O4—Zn1—O4^i^	179.999 (1)
C15—C14—C11	121.7 (3)	O5—Zn1—O6	94.07 (11)
C14—C15—C16	126.8 (3)	O5^i^—Zn1—O6	85.93 (11)
C14—C15—H15	116.6	O4—Zn1—O6	89.52 (12)
C16—C15—H15	116.6	O4^i^—Zn1—O6	90.48 (12)
O5—C16—C15	125.3 (3)	O5—Zn1—O6^i^	85.93 (11)
O5—C16—C17	114.8 (3)	O5^i^—Zn1—O6^i^	94.07 (11)
C15—C16—C17	119.9 (3)	O4—Zn1—O6^i^	90.48 (12)
C22—C17—C18	117.4 (3)	O4^i^—Zn1—O6^i^	89.52 (12)
C22—C17—C16	119.4 (3)	O6—Zn1—O6^i^	180.0

Symmetry codes: (i) −*x*, −*y*+1, −*z*+1.

### Hydrogen-bond geometry (Å, °)

**Table:**

*D*—H···*A*	*D*—H	H···*A*	*D*···*A*	*D*—H···*A*
O3—H3···O4	0.82	1.74	2.470 (3)	147
O6—H10A···O3^ii^	0.86	2.04	2.824 (4)	151

Symmetry codes: (ii) −*x*+1, −*y*+1, −*z*+1.
